# Wintering Barnacle Geese Exhibit an Increased Behavioural Drive for Sleep After Sleep Deprivation Without a Clear EEG‐Based Sleep Rebound

**DOI:** 10.1111/jsr.70221

**Published:** 2025-10-06

**Authors:** Robin Pijnacker, Giancarlo Allocca, Alexei L. Vyssotski, Peter Meerlo, Sjoerd J. van Hasselt

**Affiliations:** ^1^ Neurobiology Expertise Group, Groningen Institute for Evolutionary Life Sciences University of Groningen Groningen the Netherlands; ^2^ Florey Department of Neuroscience and Mental Health University of Melbourne Parkville Australia; ^3^ Somnivore Pty. Ltd. Parkville Australia; ^4^ Institute of Neuroinformatics Swiss Federal Institute of Technology (ETH) and University of Zurich Zurich Switzerland

**Keywords:** birds, EEG, geese, seasonal variation, sleep deprivation, sleep homeostasis

## Abstract

Sleep is essential for normal physiological functioning, and sleep deprivation is typically compensated by increasing subsequent sleep duration and/or intensity. However, a recent study showed that barnacle geese ( *Branta leucopsis* ) exhibit seasonal variation in sleep homeostasis, with full recovery of sleep after sleep deprivation in summer but no sleep rebound after similar deprivation in winter based on electroencephalography (EEG). This lack of sleep rebound could suggest that geese in winter do not build up sleep pressure during wakefulness or that accumulated sleep need is not reflected in EEG‐based sleep measures. The current study investigated whether geese in winter accumulate sleep pressure during extended wakefulness, using behavioural activity and reactivity to stimulation as alternative indicators of sleep drive. If sleep deprivation increases sleep pressure, we expected geese to adopt more sleep postures and show elevated arousal thresholds in response to stimulation. Fifteen barnacle geese were implanted with epidural electrodes for EEG recordings and housed in a semi‐natural enclosure during winter. We carefully observed and approached the geese at 10‐min intervals during the night for 8‐h following sunset. Although sleep was suppressed during this period, it did not lead to significant EEG changes and most of the lost sleep was not recovered. However, the behavioural observations revealed that geese exhibited increased sleep postures and diminished responsiveness to being approached. Our findings suggest that prolonged wakefulness in barnacle geese increases behavioural indicators of sleep pressure, also in winter, even though this rise in sleep drive is not clearly reflected in EEG‐based sleep measures.

## 1 Introduction

Sleep is a widespread phenomenon observed across a wide range of species, from jellyfish and insects to birds and mammals (Keene and Duboue 2018; Kelly et al. 2019; Libourel and Herrel 2016; Rattenborg and Ungurean 2023). This widespread occurrence suggests that sleep serves crucial functions, especially since sleeping animals cannot forage, mate, or protect themselves from predators. The importance of sleep is further emphasized by its homeostatic regulation, as evidenced by a dose‐dependent rebound sleep following sleep deprivation (Benington 2000; Deboer 2015). Lost sleep can be recovered either by extending sleep duration later on, as has been shown in various species of invertebrates and vertebrates, or by increasing subsequent sleep intensity, a response particularly well documented in mammals (Deboer 2015). The increased sleep intensity is reflected in a rise in EEG spectral power, particularly within the 1–4 Hz slow‐wave range (Deboer 2015; Vyazovskiy et al. 2011). This rise in EEG slow‐wave power has become widely accepted as an indicator of homeostatic sleep pressure or sleep drive (Dijk et al. 1987) (Franken et al. 1991; Huber et al. 2000) (Tobler and Borbely 1986).

However, while studies in mammals under laboratory conditions suggest that sleep is a tightly regulated state and that EEG spectral power reliably reflects sleep homeostasis, other studies suggest that the distribution and duration of sleep can vary under the influence of environmental factors such as photoperiod (Dijk and Daan 1989; Deboer and Tobler 1996; Kendall‐Bar et al. 2023). Particularly, research in birds has revealed significant environmentally induced variation in both sleep time and responses to sleep deprivation. For example, studies conducted under natural conditions have shown that some bird species can go without sleep for prolonged periods without clear homeostatic regulation. Great frigatebirds ( *Fregata minor* ) sleep only 0.7 h per day during six‐day foraging flights over the ocean, compared to 12 h when on land (Rattenborg et al. 2016). Similarly, male pectoral sandpipers ( *Calidris melanotos* ) were found to increase their daily activity time to 95% during the breeding season to successfully sire offspring, suggesting that sleep deprivation can even have adaptive value (Lesku et al. 2012). Also, other bird species, such as starlings (van Hasselt et al. 2020), jackdaws (van Hasselt et al. 2024) and geese (van Hasselt et al. 2021) have been shown to display large seasonal variation in sleep time.

In one of our recent studies, we found that Barnacle geese ( *Branta leucopsis* ) under semi‐natural conditions not only show seasonal variation in sleep time but also show seasonal differences in their response to sleep deprivation (van Hasselt et al. 2021). While in general the geese showed minimal changes in EEG spectral power following 4–8 h of sleep deprivation, sleep loss during summer was fully recovered by increasing subsequent sleep duration. In contrast, after 4–8 h of sleep deprivation in winter, the geese did not make up for the sleep that was lost. Such findings raise intriguing questions regarding sleep homeostasis in these birds.

There are several possible explanations for the lack of sleep rebound in winter. First, barnacle geese in the physiological winter state might accumulate sleep pressure at a much slower rate, resulting in a lower or absent need for recovery sleep. Second, barnacle geese in winter do build up sleep pressure during extended wakefulness, but the lost sleep is not recovered. Third, the sleep pressure and need for sleep that build up during extended wakefulness are recovered in a way that is not reflected in EEG‐derived sleep measures.

In the current study, we investigated whether barnacle geese in their winter state accumulate sleep pressure and an increased drive for sleep during experimentally extended wakefulness by recording their sleep–wake behaviour and assessing their behavioural responses to stimulation. While in our previous study, the lack of a sleep rebound and EEG changes following sleep deprivation suggested no increased drive for sleep, we now assessed behavioural activity and reactivity as an alternative indicator of sleep drive. If sleep deprivation increases sleep pressure in the geese, we hypothesized that they would be more likely to adopt sleep postures and show an elevated arousal threshold in response to stimulation, as has been shown in mammals (Ferrara et al. 1999; Neckelmann and Ursin 1993; Williams et al. 1964).

## 2 Methods

### 2.1 Animals and Housing

A total of 15 barnacle geese ( *Branta leucopsis* ), including both sexes and aged between 1.5 and 10 years old, were group‐housed in a semi‐outdoor aviary (10 × 4 m). All animals had unilaterally clipped flight feathers to prevent flight and were individually colour‐banded. The geese had unrestricted access to food and water (food item numbers 615,220 and 384,020; Kasper Faunafood, Woerden, The Netherlands) and were exposed to the natural light–dark cycle and climate in winter (LD 8:16). All experiments were approved by the national central authority for scientific procedures on animals (CCD) and the institutional animal welfare body (IVD, University of Groningen, the Netherlands).

### 2.2 Surgery

To measure brain activity and assess sleep–wake patterns, geese underwent surgery to implant epidural EEG electrodes. An additional electrode was placed over the neck muscle to record EMG. General anaesthesia was induced using 5% isoflurane, which was then reduced to 1%–2% after the loss of consciousness and adjusted as needed throughout the surgery. Breathing was closely monitored to ensure proper depth of the anaesthesia. A subcutaneous injection of meloxicam was administered as an analgesic (0.5 mg/kg). Moreover, following removal of feathers from the head, lidocaine was applied externally for additional pain relief (2 mg/mL). A midline incision was made on the head, and the exposed skull was cleaned of membranes. Six 0.5 mm holes were drilled, three on each hemisphere, for close fitting of rounded gold‐plated pins (BKL Electronic 10,120,538, Lüdenscheid, Germany). Three EEG electrodes were inserted in the holes in a left‐to‐right configuration: two on the left hemisphere (2 and 6 mm lateral to the midline) and one on the right hemisphere (2 mm lateral to the midline). The remaining three holes near the cerebellum were used for a ground electrode (on the midline) and two reference electrodes (4 mm lateral to the midline on each hemisphere). One reference electrode was linked to two frontal EEG electrodes that were placed over both hemispheres. The other reference electrode was linked to the third EEG electrode and EMG electrode. Moreover, a wire was placed on the neck muscle to record the EMG (PlasticsOne, Ranoke, VA, USA). The position of our electrodes was based on earlier reported studies in birds, including our own study in geese (van Hasselt et al. 2021), which allows for an effective differentiation between vigilant states and measures the slow waves that are characteristic for NREM sleep. The electrodes were soldered to a connector (BKL Electronic 10120302, Lüdenscheid, Germany) and secured with Paladur dental acrylic (Heraeus Kulzer, Hanau, Germany). When the dental acrylic hardened, a cap was attached to the implant for protection of the pins. Additional lidocaine was applied around the implant at the end of the surgery. After surgery, the animals were returned to the aviary where they could recover for at least 1 week before the first experiment.

### 2.3 Experimental Design

After the recovery period, a data logger was attached to the implant on the head of each individual (Neurologger 2A; Evolocus, Tarrytown, NY, USA). The logger recorded EEG and EMG signals as well as head movements via an onboard accelerometer (LIS302DLH; STMicro‐electronics Geneva, Switzerland) at a sampling rate of 100 Hz. Sleep–wake patterns were recorded for up to 3 days, which included a 24‐h baseline, an experimental day with 8 h of sleep deprivation followed by 16 h of recovery, and a second 24‐h recovery day. The lightweight, wireless logger allowed the geese to exhibit their normal behaviour in a semi‐natural environment.

**FIGURE 1:**
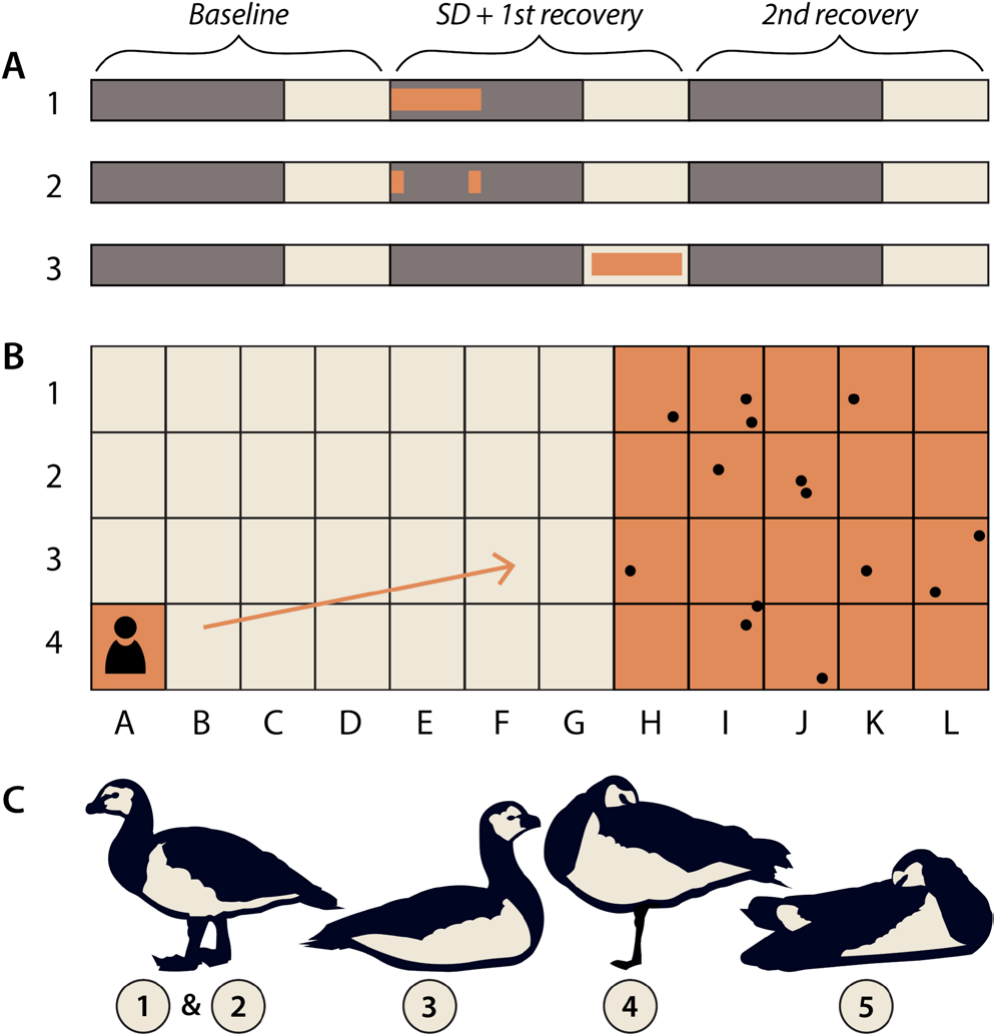
Overview of the experimental design. (A) The illustrated timeline displays three phases: Baseline, sleep deprivation (SD) with first recovery and second recovery. Dark bars indicate dark periods, while light bars represent light periods. The orange bar marks the sleep deprivation period, which lasts 8 h during the dark phase in experiment 1, includes 1 h at the beginning and 1 h at the end of the 8‐h period in experiment 2, and takes place for 8 h during the light phase in experiment 3. (B) Schematic representation of the aviary seen from above. The letters and numbers indicate the grid in which the aviary was divided. The dots show an example of the position of individual geese. In the bottom left corner, the position of the experimenter is shown. The experimenter disrupts sleep by approaching the geese, indicated by the orange arrow. The positions of the experimenter and the group of geese in the aviary are marked by the orange‐coloured areas. (C) The bottom illustration depicts the different body postures of the geese. From left to right: Active or inactive while standing, inactive sitting, head in feathers standing, head in feathers sitting. These postures are numbered from 1 to 5, with most active labelled with 1 and most sleepy labelled with 5.

During the baseline day, the geese were left undisturbed. The 8‐h sleep deprivation began at sunset of the second night (Figure 1A). During this period, we recorded behavioural postures at 10‐min intervals as a proxy for sleep. In each interval, the first minute was used to document behaviours, whereas the geese were left undisturbed for the remaining 9 min. An ethogram was created to track body posture, position within the aviary, and the minimum distance required to arouse the geese. The body postures were categorised on a scale from 1 to 5 (Figure 1C), where 1 indicated the most activity (eating, walking), 2 indicated standing still, and 3 indicated sitting without any movements for at least 5 s. Scores of 4 and 5 were given when the geese were standing or sitting with their head tucked into their feathers, a sign of deeper sleep (Dewasmes et al. 1985). The position of the geese within the aviary was tracked using a grid system visible on 3 of the 4 walls. The area of the aviary was divided into 48 squares (84 × 98 cm), arranged in 4 rows and 12 columns, marked 1–4 along the width of the aviary and A–L along the length of the aviary (Figure 1B). This grid helped to estimate the location of the geese at each 10‐min interval. When the posture and position of the geese were clear, experimenters carefully approached them. Based on our experience, we knew this was sufficient to arouse the geese. While we have no data on this, it may be that one or all the geese sleep with one eye open, which is also reported in other waterfowl (Rattenborg et al. 1999). When all geese showed clear signs of wakefulness and activity, the experimenters noted their own location in the aviary and returned to the corner (position A4, Figure 1B). The relative distance was calculated by dividing the distance the experimenter walked by the distance between the geese and the experimenter before approaching. After each hour, the starting position of the experimenters changed from A4 to L4 and vice versa to prevent habituation of the geese to humans (Figure 1B). The procedure of approaching the geese in the first minute of each 10‐min episode proved to be an effective way to keep the geese awake and resulted in a near complete sleep deprivation. After 8 h, the experimenters left the aviary. The geese's sleep–wake patterns were then measured for 40 more hours to assess recovery from the experimental intervention. On the fourth day, the data loggers were retrieved and data was processed.

After the first study described above, we conducted two additional behavioural experiments. The first was done to control for any potential circadian effect on arousal threshold by assessing body posture and distance to the flock at 10‐min intervals during only the first and eighth hour of the night. The geese were left undisturbed between these two intervals to ensure that their levels of sleep pressure at the start of the intervals were similar to those on the baseline day (Figure 1A). This approach allowed us to test whether the observed patterns in arousal threshold and body postures were driven by clock time or by sleep pressure.

The second additional experiment was done to control for the potential effect of habituation of the animals to the experimenter. With the same animals as in the first two experiments, body posture and distance to the flock were measured at 10‐min intervals for 8 h during the daytime of the second day (Figure 1A). Since in winter the geese sleep little during the daytime, any change in behaviour and responsivity would most likely be a result of habituation to the experimenters rather than a loss of sleep and increased sleep pressure.

### 2.4 Signal and Data Analyses

The data collected from the loggers were converted to standardised European Data Format (EDF) and imported into the machine learning program Somnivore for further processing (Somnivore Pty. Ltd., Parkville, VIC, Australia). This program used a subset of manually scored epochs per recording to classify wakefulness, NREM sleep, and REM sleep episodes. NREM sleep epochs were characterised by low‐frequency, high‐amplitude EEG signals, low EMG activity, and minimal accelerometer output. In contrast, REM sleep epochs were marked by high‐frequency, low‐amplitude EEG signals, muscle atonia, and the absence of clear head movements, except for occasional head drops or head wobbling that could be detected in the accelerometry traces. These criteria for REM sleep scoring are commonly used in avian sleep literature. Lastly, wakefulness was indicated by high‐frequency, low‐amplitude EEG signals coinciding with higher EMG signals and frequent head movements. See Figure S1 for representative power spectra of the baseline vigilant states in barnacle geese as published in our previous study (van Hasselt et al. 2021; Figure S1). The program integrates manual scoring data with features from the EEG, EMG and accelerometer channels to accurately determine sleep stages every 4‐s epoch. A minimum of 100 epochs for each stage was manually scored before applying Somnivore's automated EEG classification. This way of scoring has been validated for multiple bird species including pigeons (Allocca et al. 2019) and geese (van Hasselt et al. 2021). For geese, the automated scoring had an accuracy of 0.98 ± 0.01 for wakefulness, 0.97 ± 0.01 for NREM sleep, and 0.84 ± 0.04 for REM sleep as compared to the manual scoring.

After the initial autoscoring, the recordings were inspected for artefacts. An epoch was classified as an artefact when the EEG signal amplitude was at least twice as high as that observed during normal sleep stages. These artefacts mainly occurred during wakefulness and did not affect the sleep stage classification, but were filtered out to prevent contaminating the spectral power analysis. The percentage of artefacts identified was 25.4% ± 3.4% during wakefulness. The EEG signals of animals during rest and sleep were generally of good quality and did not contain artefacts this much, 4.2% ± 2.7% during NREM sleep, and 1.5% ± 0.9% during REM sleep. The average number of NREM and REM sleep epochs per hour was calculated. Additionally, a Fast Fourier transformation (FFT) was performed on the artefact‐free NREM sleep epoch, resulting in 256 frequency bands with a bin width of 0.19 Hz. To account for individual differences in absolute EEG power, the NREM sleep EEG power for each frequency band was normalised relative to the mean power during the 24‐h baseline day in the same band. The 256 frequency bands were subsequently averaged into three bands that are frequently used in sleep research: Delta (1.56–3.91 Hz), Theta (3.91–8.20 Hz) and Alpha (8.20–14.06 Hz) (van Hasselt et al. 2021). While it is unsure whether these specific bands have a functional relevance in birds, we included them for comparative reasons, and they are used in our previous study.

### 2.5 Statistics

**FIGURE 2:**
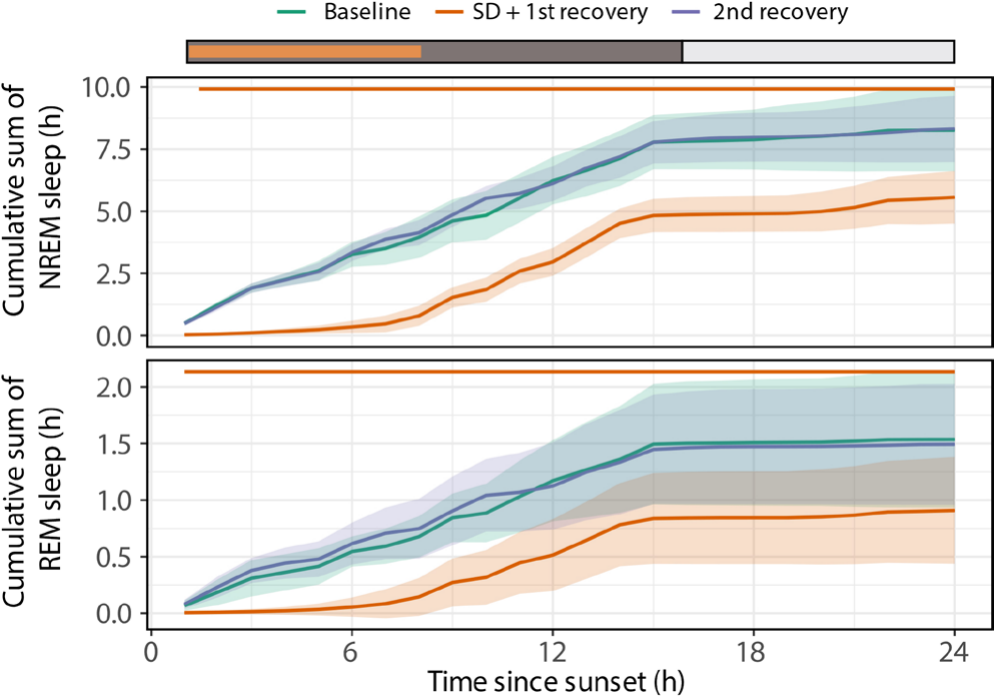
Effect of sleep deprivation on NREM and REM sleep patterns and cumulative sleep time (*n* = 6). Sleep deprivation began following sunset and lasted 8 h, marked by the orange bar at the bottom of the graph. The dark bar represents the dark phase, and the white bar represents the light phase. Each panel displays sleep during baseline (green line), the SD period and immediate recovery (orange line), and the extended recovery period (blue line). Data are shown as group averages ± SEM. The accumulated NREM and REM sleep were significantly reduced during each hour of SD and the recovery period compared to baseline (Hour 24: REM, *p* < 0.0001; NREM, *p* < 0.0001, lmer model).

**FIGURE 3:**
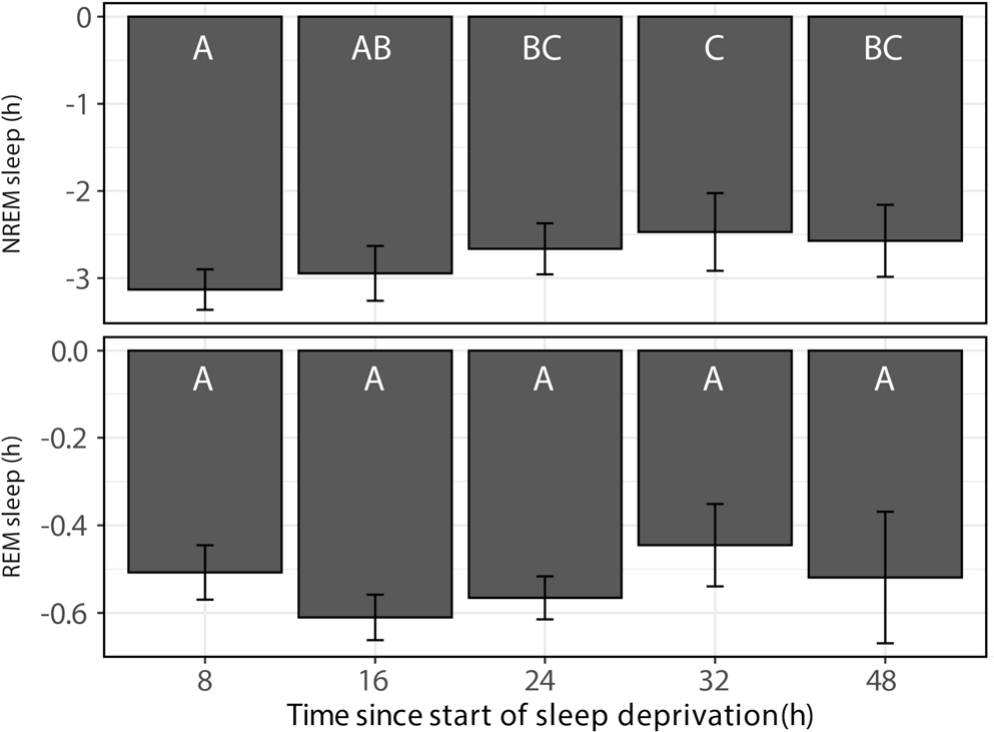
Difference in cumulative NREM and REM sleep time between baseline and recovery days. A negative difference in sleep time indicates a loss of sleep after sleep deprivation, compared to baseline. The first bar indicates the sleep lost at the end of the 8 h sleep deprivation. The subsequent bars indicate the sleep loss that remained during the recovery period thereafter. Bars that share the same letter (A, B, or C) are not statistically different from each other. Although there is a slight and significant reduction in NREM sleep loss, the sleep deprivation is not fully compensated (*p* < 0.05, post hoc test after lmer model). No significant decrease in the amount of lost REM sleep can be observed. Data shown are group averages ± SEM.

**FIGURE 4:**
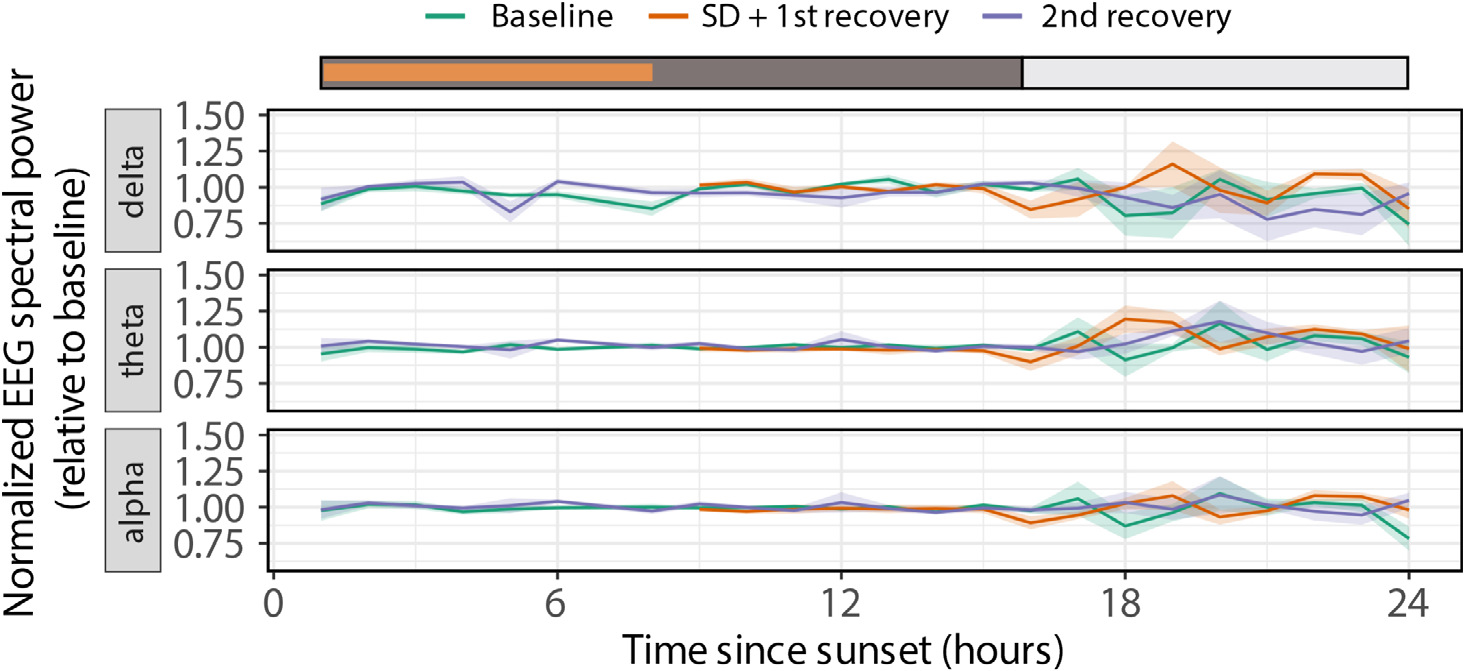
NREM sleep EEG power (relative to average baseline) over time, for Baseline (green), SD + 1st recovery (orange), and 2nd recovery (blue) (*n* = 6) for the analysed frequency bands (Delta: 1.56–3.91 Hz; Theta: 3.91–8.20 Hz; Alpha: 8.20–14.06 Hz). The coloured bar on the top of the graph represents the dark phase (dark grey), light phase (white) and the sleep deprivation episode (orange). There is no increase in NREM sleep EEG power after sleep deprivation. Data presented are group averages ± SEM.

**FIGURE 5:**
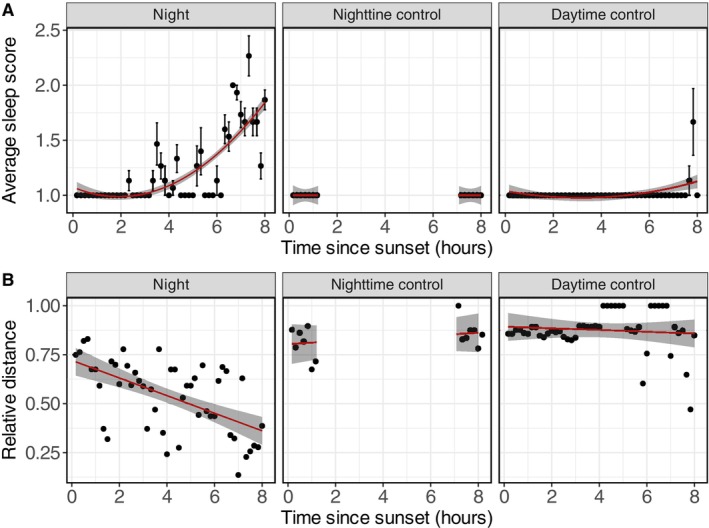
Changes in behavioural measurements during sleep deprivation. (A) Effects of sleep deprivation on sleep score when sleep deprivation was performed for 8 h at night (left panel), for 1 h at the start and end of the 8 h at night (middle panel), or for 8 h during the day (right panel). Sleep score significantly increases during 8 h of sleep deprivation at night, but not during the other sleep deprivation protocols. (B) Effects of sleep deprivation on the relative distance between experimenter and geese. Sleep deprivation only decreases the relative distance when performed at night for eight consecutive hours. Averaged data are shown as mean ± SEM. Model output is shown as the model fit +95% CI).

Data were analysed in R. For the EEG‐based results (Figures 2, 3, 4), data were analysed using linear mixed effect models as implemented by the lme4 package with bird ID as a random effect. The behavioural results (Figure 5) were analysed using a linear regression model using the same lme4 package. Post hoc analyses were performed using the lsmeans package. Data in the text and in Figures 2, 3, 4 are expressed as mean ± SEM. Data in Figure 5 are expressed as model fit ±95% confidence interval (CI).

## 3 Results

The geese exhibited a clear daily rhythm in sleep and wakefulness, with 93% of their baseline sleep occurring at night and only 7% during the day. On average, the geese spent 59.1% ± 4.7% of the 24‐h period awake, 6.4% ± 1.3% in REM sleep and 34.3% ± 3.5% in NREM sleep. The average duration of sleep bouts was 15.5 ± 7.3 s for NREM sleep and 5.6 ± 1.2 s for REM sleep. The minimum bout duration for both sleep states was 4 s, whereas the maximum bout duration for NREM sleep and REM sleep was 7.5 ± 3.8 min and 1.16 ± 0.5 min, respectively. These values on sleep time and sleep bout duration are in line with values reported by van Hasselt et al. (2021).

The geese were subjected to an 8‐h protocol during which they were approached once every 10 min, which was sufficient to arouse them from sleep, perhaps because some animals sleep with one eye open (Rattenborg et al. 1999). Geese are highly sensitive to the presence of the experimenters, although the animals were not disturbed during the remainder of each 10‐min episode. Figure 2 demonstrates that sleep deprivation was successful. There was significantly less sleep during the first 8 h of the SD + 1st recovery day compared to the baseline. The geese lost 3.1 ± 0.2 h of NREM sleep and 0.5 ± 0.1 h of REM sleep. In the first 5–6 h, the geese lost all sleep they normally have under baseline conditions. Only towards the end of the sleep deprivation period did the geese show a slight increase in sleep time. During the second recovery day, there was no increase in NREM and REM sleep compared to baseline, suggesting that the sleep lost during the sleep deprivation period was not fully recovered. Sleep bout duration during the first 2 h after recovery was not different compared to the same hours during baseline, for NREM sleep 16.6 ± 7.6 versus 19.2 ± 8.5 s respectively and for REM sleep 5.8 ± 1.4 versus 5.6 ± 1.4 s respectively (posthoc test after LMER model; *p* = 0.40 and *p* = 0.11, respectively for NREM and REM sleep). These results are in line with values reported by van Hasselt et al. (2021).

To assess how much of the sleep loss was recovered within 2 days, we calculated the difference in cumulative sleep time between baseline and various time points after sleep deprivation, as shown in Figure 3. Negative values for different time points indicate a remaining sleep deficit. After 48 h from sunset (40 h of recovery), there was a slightly but significantly smaller NREM sleep deficit than immediately after sleep deprivation (time 8–48, *p* = 0.0122). However, after 40 h of recovery, both NREM and REM sleep deficits remained, as cumulative sleep differences do not return to baseline.

We also calculated the average EEG power for three frequency bands, as depicted in Figure 4. During baseline night, EEG power remained relatively constant over the 15 h of sleep. The typical pattern of increased delta power at the start of a sleep period, followed by a decrease over the night, as seen in many other species, was not observed here. Also, after the 8 h of sleep deprivation, there was no immediate response to the extended wake period. The EEG power did not differ significantly from the baseline night for any of the three frequency bands. Also, during the recovery day after sleep deprivation, no significant changes in EEG power were observed. Overall, no clear effect of sleep deprivation on EEG power was evident.

During the 8 h nighttime sleep deprivation, the geese increasingly displayed sleep‐like behaviours, as shown by a decline in active individuals and an increase in the number of geese standing still, sitting or tucking their heads under their feathers. Figure 5A shows a significant increase in sleep scores during nighttime sleep deprivation. In contrast, no significant effect on sleep scores was observed when sleep deprivation occurred during the day or was limited to the beginning and end of the night. Additionally, experimenters had to move progressively closer to the geese over the course of the night to maintain their wakefulness, suggesting increased arousal difficulty (Figure 5B). For the other conditions, the relative distance to arouse the geese did not significantly change with repeated approaches.

## 4 Discussion

In a previous study, we found that barnacle geese do not show a sleep rebound when subjected to sleep deprivation in winter (van Hasselt et al. 2021). This might suggest that the birds either do not accumulate sleep pressure during prolonged wakefulness, or that the build‐up of sleep need and its subsequent recovery are not captured by EEG‐based sleep measures. To further explore whether wintering barnacle geese experience an increased sleep drive after sleep deprivation, the current study recorded behavioural measures of sleep drive in addition to EEG. The EEG recordings showed that the sleep deprivation successfully suppressed sleep for almost the entire 8‐h experiment. Only in the last 1–2 h was there a slight increase in sleep propensity. Our behavioural observations revealed that after 8 h of sleep disruption, the geese exhibited a reduction in active postures, an increase in sleep postures, and a diminished responsiveness to being approached by human experimenters.

One might argue that such a reduction in activity and arousability in the course of the night could reflect a circadian or time‐of‐day effect rather than a result of the sleep loss that was induced. However, when we assessed body postures and arousal thresholds only during the 1st and 8th hour of the night, without disrupting sleep during the 7 h in between, we did not observe the increase in sleep behaviour.

Another possible explanation for the reduction in activity and arousability over the course of the night could be that the geese were habituating to the presence of the experimenters and the procedures to disrupt their sleep. To test this possibility of habituation, we subjected the geese to a similar 8‐h stimulation protocol during the daytime. Since the geese in winter sleep little during the day, any change in behavior and responsiveness would support the idea of habituation to the experimenters rather than an increase in sleep pressure resulting from sleep loss. However, the results of this control experiment showed no changes in behavior or reduction in responsiveness. In fact, the level of activity and responsiveness remained consistently high throughout the day, similar to the levels observed at the start of the nighttime experiment before sleep disruption.

Thus, the experiments together favor the interpretation that the reduction in activity and arousability following 8 h of stimulation during the night was a consequence of sleep loss and a concomitant increase in sleep drive, even though this rise in sleep drive was not clearly followed by a sleep rebound based on EEG recordings. Consistent with previous research (van Hasselt et al. 2021), our EEG data showed no substantial recovery of sleep lost during the deprivation night. While there was a slight and delayed increase in sleep duration the day after the sleep disruption, this accounted for only a fraction of the lost sleep. Moreover, there was no increase in EEG spectral power after sleep deprivation, which is typically considered a marker of sleep intensity (Deboer 2015). This raises two possibilities: first, there may have been an increased need for sleep that was not actually recovered, or second, the need for sleep may have been recovered but not reflected in sleep duration or EEG spectral power.

The possibility that barnacle geese accumulate a need for sleep during extended wakefulness but do not recover the lost sleep could mean these animals either lack the ability to recover sleep or somehow tolerate a loss of sleep. The latter explanation aligns with findings in other bird species, such as frigatebirds that sleep for less than an hour a day during six‐day foraging flights over the ocean (Rattenborg et al. 2016), or male pectoral sandpipers that hardly get any sleep during the breeding season yet still successfully sire offspring (Lesku et al. 2012). But also in marine mammals, such as fur seals, show no clear rebound to extended periods of sleep suppression when being in water (Lyamin et al. 2018). These studies may suggest that, under certain conditions, sleep deprivation can even have adaptive value (Lesku and Rattenborg 2022; Zaid et al. 2024). The question that remains then is what the adaptive value of tolerating sleep loss could be in wintering barnacle geese.

Alternatively, the idea that functional recovery from sleep deprivation in barnacle geese may occur without it being reflected in typical EEG changes and sleep rebound deserves attention. In the geese, EEG spectral power during sleep was stable throughout the night and showed no significant changes after sleep deprivation. This contrasts with findings in mammals, where EEG power during sleep generally increases as a function of prior wake duration (Dijk et al. 1987) (Franken et al. 1991; Huber et al. 2000) (Tobler and Borbely 1986). Specifically, spectral power in the 1–4 Hz delta range typically peaks at the start of the main sleep phase, declines in the course of sleep, and is elevated after sleep deprivation (Dijk et al. 1987) (Franken et al. 1991; Huber et al. 2000) (Tobler and Borbely 1986). Partly similar patterns have been reported in a number of bird species (e.g., Johnsson et al. 2022; Martinez‐Gonzalez et al. 2008; van Hasselt et al. 2020), although the increase in EEG power following sleep deprivation is often broader than the slow‐wave range and does not always clearly reflect the duration of prior wakefulness (van Hasselt et al. 2020). However, while most birds have some degree of EEG power increase following sleep deprivation, our barnacle geese had none.

The finding that sleep EEG spectral power in birds, and in barnacle geese in particular, is less influenced by prior wakefulness may suggest that the avian brain has a more limited capacity to increase EEG power. This might be due to the structural differences between the avian and mammalian brains (van der Meij et al. 2019; Rattenborg et al. 2009). In mammals, the brain has a layered cortical sheet, whereas in birds, large parts of the brain are organised in nuclear structures (Jarvis et al. 2013). As a result, the accumulation of sleep pressure and the subsequent sleep‐related recovery processes that occur at the cellular or network level may not manifest in the bird EEG in exactly the same way they do in most mammals.

Given that our wintering barnacle geese exhibited an increased behavioural drive for sleep after sleep deprivation but did not exhibit a clear sleep rebound, it remains uncertain whether they accumulated a sleep debt that was not recovered or whether they had a recovery response that was not reflected in the EEG. It might be worth exploring whether sleep deprivation in geese has functional consequences similar to those often reported in mammals, for example, reduced performance on various cognitive tasks (Havekes et al. 2015; Kreutzman et al. 2015). Additionally, given the seasonal variation in sleep homeostatic responses to sleep deprivation in barnacle geese, it would be interesting to assess whether the functional consequences of sleep deprivation vary with season as well.

## Author Contributions

R.P., S.J.V.H., and P.M. conceived the project and designed the experiment. R.P. and S.J.V.H. executed the experiment and analyzed the data. G.A. and A.L.V. provided technical and analytical support. The manuscript was written by R.P., S.J.V.H., and P.M.

## Conflicts of Interest

Author Giancarlo Allocca (GA) is affiliated with Somnivore Pty. Ltd., Parkville, VIC, Australia.

## Supporting information


**Data S1:** jsr70221‐sup‐0001‐Supinfo.docx.

## Data Availability

The data that support the findings of this study are available from the corresponding author upon reasonable request.
