# Supplementary material for: Wintering Barnacle Geese Exhibit an Increased Behavioural Drive for Sleep After Sleep Deprivation Without a Clear EEG‐Based Sleep Rebound
Source: J Sleep Res. 2025 Oct 6;35(3):e70221. doi: 10.1111/jsr.70221 (PMC13193403; doi:10.1111/jsr.70221)
Supplement: Supplementary file 1 — Data S1: jsr70221‐sup‐0001‐Supinfo.docx. [file JSR-35-e70221-s001.docx]

**Wintering barnacle geese exhibit an increased behavioural drive for sleep after sleep deprivation without a clear EEG-based sleep rebound**

Robin Pijnacker^1^, Giancarlo Allocca^2,3^, Alexei L. Vyssotski^4^, Peter Meerlo^1#^, Sjoerd J. van Hasselt^1#^

^1^ Neurobiology Expertise Group, Groningen Institute for Evolutionary Life Sciences, University of Groningen, Groningen, The Netherlands

^2^ Florey Department of Neuroscience and Mental Health, University of Melbourne, Parkville, VIC 3010, Australia

^3^ Somnivore Pty. Ltd., Parkville, VIC 3340, Australia

^4^ Institute of Neuroinformatics, Swiss Federal Institute of Technology (ETH) and University of Zurich, Switzerland

^#^ These authors contributed equally

**Corresponding author**

Peter Meerlo

Neurobiology Expertise Group

Groningen Institute for Evolutionary Life Sciences

University of Groningen

Nijenborgh 7

9747 AG Groningen

The Netherlands

p.meerlo@rug.nl

Figure S1. Averaged absolute EEG power based on artifact-free epochs of the three vigilant states of barnacle geese recorded in winter (blue) and summer (red). This figure is adapted from Van Hasselt et al., 2021.

Reference:

van Hasselt, S. J., Mekenkamp, G.-J., Komdeur, J., Allocca, G., Vyssotski, A. L., Piersma, T., Rattenborg, N. C., Meerlo, P. (2021). Seasonal variation in sleep homeostasis in migratory geese: a rebound of NREM sleep following sleep deprivation in summer but not in winter. Sleep, 44, zsaa244. Doi: 10.1093/sleep/zsaa244.
